# Prevalence of high blood pressure measured in the Brazilian population, National Health Survey, 2013: Prevalência da hipertensão arterial aferida na população brasileira, Pesquisa Nacional de Saúde, 2013

**DOI:** 10.1590/1516-3180.2015.02090911

**Published:** 2015-03-17

**Authors:** Deborah Carvalho Malta, Nadir Baltazar dos Santos, Rosângela Durso Perillo, Célia Landmann Szwarcwald

**Affiliations:** 1 MD, PhD. Professor and Researcher, Department of Mother and Child and Public Health, Nursing School, Universidade Federal de Minas Gerais, Belo Horizonte, MG, Brazil.; 2 BSc. Statistician, Instituto Brasileiro de Geografia e Estatística (IBGE), Rio de Janeiro, RJ, Brazil.; 3 MSc. Nurse, Municipal Health Department, Belo Horizonte, and Researcher, School of Medical Sciences, Universidade Federal de Minas Gerais, Belo Horizonte, MG, Brazil.; 4 PhD. Professor and Researcher, Institute of Health Communication and Scientific and Technological Information, Fundação Oswaldo Cruz, Rio de Janeiro, RJ, Brazil.

**Keywords:** Hypertension, Health surveys, Risk factors, Chronic disease, Cardiovascular disease

## Abstract

**CONTEXT AND OBJECTIVE:**

High blood pressure (hypertension) is the most frequent cause of morbidity and a major risk factor for cardiovascular complications. The aim here was to describe the prevalence of blood pressure greater than or equal to 140/90 mmHg in the adult Brazilian population and federal states, along with self-reported information about previous medical diagnoses of hypertension, use of medication and medical care for hypertension control.

**DESIGN AND SETTING:**

Cross-sectional study analyzing information from the National Health Survey of 2013, relating to Brazil and its federal states.

**METHODS:**

The sample size was estimated as 81,254 households and information was collected from 64,348 households. The survey consisted of interviews, physical and laboratory measurements. Systolic blood pressure was considered to be high when it was ≥ 140 mmHg and diastolic blood pressure, ≥ 90 mmHg.

**RESULTS:**

It was found that 22.8% of the population has blood pressure measurements ≥ 140/90 mmHg. The proportion was higher among men than among women: 25.8% versus 20.0%. The frequency increased with age, reaching 47.1% in individuals over 75 years and was highest in the southeast and south. 43.2% reported previous medical diagnoses of hypertension and, of these, 81.4% reported using medication for hypertension and 69.6%, going to the doctor within the past year for pressure monitoring, thus showing regular medical follow-up.

**CONCLUSION:**

These results are important for supporting measures for preventing and treating hypertension in Brazil, with the aim of achieving the World Health Organization's goal of reducing hypertension by 25% over the next decade.

## RESUMO

**CONTEXTO E OBJETIVO:**

Pressão alta (hipertensão) é a causa mais frequente de morbidade e importante fator de risco para complicações cardiovasculares. O objetivo foi descrever a prevalência de pressão arterial maior e igual a 140/90 mmHg na população adulta brasileira e nas Unidades Federadas, bem como informações autorreferidas sobre diagnóstico médico prévio de hipertensão, uso de medicação e acompanhamento médico para controle de hipertensão arterial.

**TIPO DE ESTUDO E LOCAL:**

Estudo transversal, analisando informações da Pesquisa Nacional de Saúde em 2013, referentes ao Brasil e Unidades Federadas.

Métodos: A amostra foi estimada em 81.254 domicílios e foram coletadas informações em 64.348 unidades domiciliares. A PNS consistiu em entrevistas, medidas físicas e laboratoriais. A pressão arterial foi considerada elevada quando a sistólica aferida ≥ 140 mmHg ou a pressão arterial diastólica ≥ 90 mmHg.

**RESULTADOS:**

Foi identificado que 22,8% da população tem pressão arterial medida ≥ 140/90 mmHg, sendo mais elevada em homens 25,8%, e 20,0% em mulheres. A frequência aumenta com a idade, chegando a 47,1% acima de 75 anos e as medidas foram mais elevadas nas regiões Sudeste e Sul; 43,2% referiram diagnóstico médico prévio de hipertensão; destes, 81,4%, relataram usar medicação para hipertensão e 69,6% foram ao médico no último ano para monitoramento da hipertensão, mostrando acompanhamento médico regular.

**CONCLUSÃO:**

Estes resultados são importantes para apoiar medidas de prevenção e tratamento ao hipertenso no país, visando atingir a meta da Organização Mundial de Saúde de redução da hipertensão em 25% na próxima década.

## INTRODUCTION

High blood pressure is the most frequent cause of morbidity and is the main risk factor for cardiovascular complications such as stroke, acute myocardial infarction, chronic kidney disease and vascular diseases, among others.^1^^,^^2^ It is one of the causes of decreased quality of life and life expectancy and leads to high socioeconomic costs, thereby directly affecting individuals, families, the healthcare system and the economy.^2^^,^^3^

The World Health Organization (WHO) has estimated that approximately 25% of the world population has high blood pressure, and growth of 60% in the numbers of cases of this disease by 2025 has been predicted.^1^^,^^2^ In Brazil, cardiovascular diseases are responsible for 30% of deaths with known causes, and are also the biggest cause of hospitalization in the Brazilian National Health System (SUS).^4^^,^^5^

Because of operational difficulties and the high cost of measuring high blood pressure in the field, studies using self-reported data have been used as a proxy for these measurements.^6^^,^^7^ In Brazil, health surveillance done by means of telephone interviews (VIGITEL surveys) have indicated that the overall prevalence of high blood pressure is 24.8%, and that the prevalence increases with age, such that more than half the population over the age of 55 years is affected.^8^

Population-based prevalence studies on blood pressure measurements in Brazil are still scarce. Most of them are limited to institutions or municipalities, without national scope. Different methodological approaches have been used, along with different samples, different population groups (sex, age, income and educational level) and different diagnostic criteria, without any standardization when measuring blood pressure, which makes comparisons difficult.^3^^,^^9^

A review study that used blood pressure of 140/90 mmHg as a cutoff indicated prevalence of around 20%, without distinction between the sexes, but with an evident tendency towards increasing pressure with age.^3^ In Brazil, population-based studies have revealed that between 37% and 67% of patients with high blood pressure are treated, but that blood pressure control is low among these treated patients, reaching levels of only 20% to 26%.^9^

The Longitudinal Study of Adult Health (Estudo Longitudinal de Saúde do Adulto, ELSA-Brasil) used a cohort composed of teachers and other employees at six Brazilian universities aged between 35 and 74 years. The study demonstrated that 35.8% of the participants met the predefined criteria for hypertension, which were systolic/diastolic blood pressure greater than or equal to 140/90 mmHg or use of medication prescribed for high blood pressure. Moreover, among the individuals with high blood pressure, 80% were aware that they presented high blood pressure, 78% were undergoing treatment and 56% presented controlled levels.^10^^,^^11^

In 2013, the National Health Survey (Pesquisa Nacional de Saúde, PNS) included blood pressure measurement among adults, along with questions about high blood pressure diagnosed by doctors, the care provided and use of medications, among other topics.^12^^,^^13^ The present study is the first to analyze the high blood pressure data measured through the PNS in Brazil.

## OBJECTIVE

The objective of this study was to describe the prevalence of high blood pressure above 140/90 mmHg among the adult Brazilian population and within each federal state, along with self-reported information about previous medical diagnoses of hypertension, use of medication and medical follow-up for high blood pressure control.

## METHODS

This was a cross-sectional study carried out using secondary data from the PNS, which was a population-based survey conducted by the Brazilian Institute for Geography and Statistics (Instituto Brasileiro de Geografia e Estatística, IBGE) in 2013, in partnership with the Brazilian Ministry of Health. ^12^^,^^13^ The survey was household-based and the sample that was used was grouped in three stages (cluster sampling), with stratification of the primary sampling unit (PSUs). Census tracts or sets of census tracts formed the PSUs, households were the second-stage units and inhabitants aged 18 years or over were the third-stage units. The subsample of PSUs was selected through simple random sampling.

The sample size was estimated in the PNS as 81,357 households, and information was collected from 64,348 of them. Taking into account closed households, the loss rate was 20.8% and the no-response rate was 8.1%.^12^^,^^13^ A total of 60,202 people participated in the individual interviews. Among these, 59,402 people had their blood pressure measured, of whom 25,920 were men and 33,482 were women. This was the first national survey on blood pressure levels among adults aged 18 years or over. High blood pressure was taken to be a systolic blood pressure measurement of greater than or equal to 140 mmHg or a diastolic measurement of greater than or equal to 90 mmHg.^14^

Blood pressure was measured by a trained team using a calibrated digital device. The individuals needed to be at rest and were instructed to empty their bladders, not to smoke or drink during the 30-minute period preceding the measurement and not to do any physical activities during the one-hour period preceding the measurement. The blood pressure measurements were made with the individual in a seated position, having rested for at least five minutes beforehand. The subjects were instructed to keep their back relaxed and supported against the backrest of the chair, not to cross their legs and to leave their left arm free of clothing and resting on a table at the same level as their chest or heart. Three blood pressure measurements were made, with two-minute intervals between them. The measurements were then inserted into a smartphone. The average between the second and third measurements was used for the present study.^14^

A controlled imputation process was used in relation to situations that were deemed to be no-response situations. A set of integrated computational routines were used within a system named CIDAQ (Crítica e Imputação de Dados Quantitativos, i.e. Criticism and Imputation of Quantitative Data). This takes into consideration the combined behavior of all the variables recorded: age, sex, weight, height and family per-capita income. More details can be seen in other published materials.^12^^,^^14^

The present study describes the prevalence of individuals with blood pressure ≥ 140/90 mmHg in the Brazilian adult population, according to sex, age group, region of the country and federal state, with the 95% confidence interval (CI). Moreover, the following proportions were also calculated: a) people aged 18 years or over with blood pressure ≥ 140/90 mmHg at the time of the survey, according to any previous medical diagnosis of hypertension (yes or no); b) people aged 18 years or over with blood pressure ≥ 140/90 mmHg who reported having used medications for controlling high blood pressure over the past 15 days (yes or no); and c) people aged 18 years or over with blood pressure ≥ 140/90 mmHg who reported having gone to a doctor because of hypertension over the past year (yes or no).

The survey was approved by the National Ethics Commission for Research Involving Human Beings, of the Ministry of Health, under report number 328,159 of June 26, 2013. The free and informed consent statement was signed in the smartphone itself during the PNS.

## RESULTS

The analysis on high blood pressure at the time of measurement within the PNS in 2013, i.e. systolic blood pressure ≥ 140 mmHg and diastolic blood pressure ≥ 90 mmHg, indicated that the prevalence of people with high blood pressure was 22.8%.

It was found that 20.0% (95% CI: 19.3-20.8) of the women had high blood pressure, while the prevalence among men was 25.8% (95% CI: 24.8-26.7). The frequency of high blood pressure increased with age, for both sexes, reaching around 47% among people aged 75 years or over. Between the ages of 18 and 74 years, men presented higher blood pressure than women, but the prevalences became equal after the age of 75 years (Table 1).

**Table 1:**
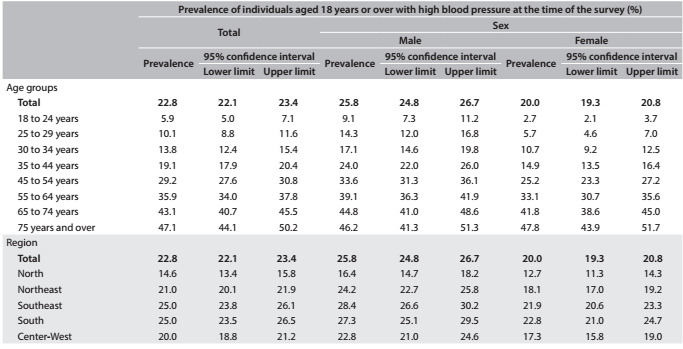
Prevalence of individuals with high blood pressure in the adult population according to age group, region and sex, in the National Health Survey (Pesquisa Nacional de Saúde, PNS), 2013

The frequencies were higher in the southeastern and southern regions of Brazil for both men and women (Table 1). In all the federal states, the frequency of people with blood pressure ≥ 140/90 mmHg was greater among men. The prevalence among the adult population ranged from 13.3% in Amazonas to 27.6% in Rio Grande do Sul (Table 2).

**Table 2:**
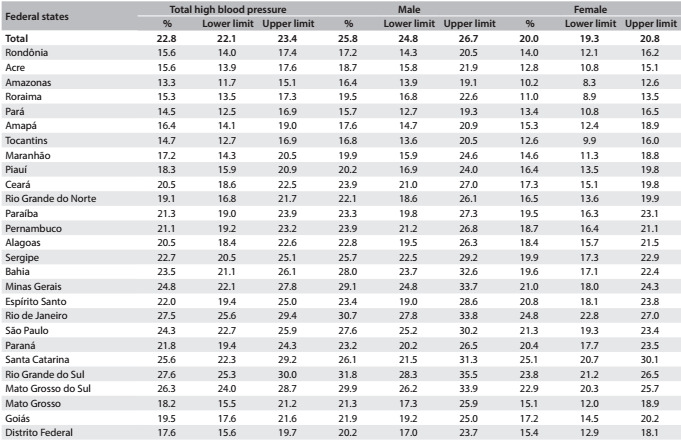
Prevalence of individuals with high blood pressure among the total number of individuals aged 18 years or over in each federal state, according to sex, in the National Health Survey (Pesquisa Nacional de Saúde, PNS), 2013

**Figure 1:**
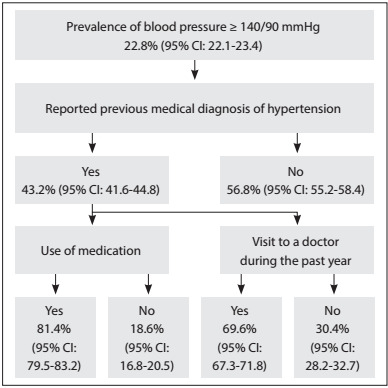
Flow diagram for the whole population with blood pressure ≥ 140/90 mmHg, according to previous medical diagnosis, use of medication and consultations with a doctor during the past year, in the National Health Survey (Pesquisa Nacional de Saúde, PNS), 2013.

Among the individuals who presented high blood pressure (≥ 140/90 mmHg), 43.2% (95% CI: 41.6-44.8) reported having a previous diagnosis of hypertension, while 56.8% did not know that they had high blood pressure. Among those with a previous medical diagnosis of hypertension, 81.4% (95% CI: 79.5-83.2) were using medication, and 69.6% (95% CI: 67.3-71.8) had visited a doctor during the past year (Figure 1).

**Figure 2:**
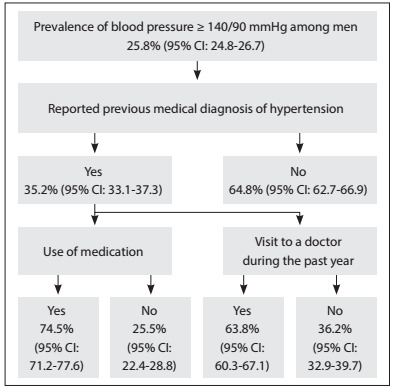
Flow diagram for the male population with blood pressure ≥ 140/90 mmHg, according to previous medical diagnosis, use of medication and consultations with a doctor during the past year, in the National Health Survey (Pesquisa Nacional de Saúde, PNS), 2013.

These percentages differed according to sex. Women presented greater occurrence of previous medical diagnoses of hypertension (52.6%; 95% CI: 50.6-54.7) than men (35.2%; 95% CI: 33.1-37.3). Women also used more medication for hypertension (86.8%; 95% CI: 84.8-88.7) than men (74.5%; 95% CI: 71.2-77.6) and visited a doctor more often (74.1%; 95% CI: 71.4-76.7) than men (63.8%; 95% CI: 60.3-67.1) (Figures 2 and ^3^).

**Figure 3:**
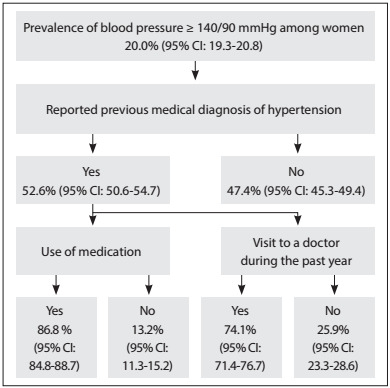
Flow diagram for the female population with blood pressure ≥ 140/90 mmHg, according to previous medical diagnosis, use of medication and consultations with a doctor during the past year, in the National Health Survey (Pesquisa Nacional de Saúde, PNS), 2013.

## DISCUSSION

This was the first national study indicating results from blood pressure measurements among the Brazilian population. One fourth of the male adult population presented blood pressure ≥ 140/90 mmHg, while high blood pressure affected one fifth of the female adult population. Its frequency increased with age, reaching almost half of the elderly population (over 75 years). Blood pressure levels were higher in the southeastern and southern regions of the country, among both men and women. In all the federal states, blood pressure ≥ 140/90 mmHg occurred more frequently among men. The lowest prevalence was found in the state of Amazonas, while the highest was observed in the state of Rio Grande do Sul. Nearly half of the population reported having previous medical diagnoses of hypertension and, out of this portion, over three quarters reported using medication for high blood pressure and close to two thirds had visited a doctor during the past year for their high blood pressure to be monitored, thus demonstrating that they were receiving regular medical follow-up. Women used more medication than men and went to a doctor more often for their high blood pressure to be monitored.

The PNS provided nationwide blood pressure measurement in Brazil for the first time, using methodology indicated in the literature. Use of digital electronic devices is widely recommended for population-based surveys, given that they reduce measurement errors and make it easier to interpret and standardize the results.^14^^,^^15^

A review study involving 35 developing countries identified 204 articles and, similarly to PNS, found greater mean prevalence of hypertension among men, reaching 32.2%, than among women, 30.5%.^16^ Greater frequency of high blood pressure among men is concordant with the data from the World Health Organization (WHO) for the year 2008, which demonstrated that among adults over the age of 25 years, the overall prevalence is higher among men (29.2%) than among women (24.8%).^1^ The same was found solely in the Americas, with 26.3% for men and 19.7% for women.^1^

There are divergences in the results regarding differences between the sexes, which may occur due to factors such as educational level, race/skin color, obesity, seeking of health services and adherence to treatment, among others.^17^ A cross-sectional study on a population-based sample of 1,439 adults ≥ 20 years of age, in Salvador, Brazil, found the opposite: greater prevalence of hypertension among women (31.7%; 95% CI: 28.5-34.9) than among men (27.4%; 95% CI: 23.9-31.2). It also reported that the factors associated with hypertension among women were mixed or black skin color, abdominal obesity, diabetes and the menopause.^17^ In the literature, higher blood pressure among women after the menopause is partly attributed to the protective hormonal effect that estrogen confers on women during their fertile phase, which stops after the menopause. Another explanatory factor for increasing hypertension with increasing age might be central obesity.^17^

The PNS also confirmed what had already been described in the literature, regarding increased prevalence of high blood pressure with age. This higher prevalence is due to the changes inherent to growing older, with greater stiffening of the arteries, greater peripheral vascular resistance and comorbidities among the elderly.^17^^,^^18^^,^^19^^,^^20^

The present study showed regional differences according to federal state and region, with greater prevalence in the southeast and south. In addition to demographic factors such as the greater participation of elderly individuals in these regions, other risk factors need to be better studied.^21^

The highest frequency among the federal states was in Rio Grande do Sul, followed by Rio de Janeiro, which can be explained because they are states with large proportions of elderly people;^22^ while the lowest was in Amazonas. A previous study carried out within the Pró-Saúde movement at a university in Rio de Janeiro, in 2001, found that the prevalence for both sexes was 29.6% and that it increased significantly with age.^23^ On the other hand, the lower prevalence observed in Amazonas can be explained by the lower proportion of elderly individuals in this state and the greater proportion of young people, as indicated in the demographic census.^22^

Studies have indicated that detection, treatment and control of high blood pressure are fundamental for reducing the incidence of cardiovascular events.^17^^,^^18^^,^^19^ The present study found that 43.2% of the subjects had previously received a medical diagnosis of high blood pressure. The proportion was higher among women, who also had greater frequencies of use of medication and routine medical consultations. One reason why almost half the population with blood pressure ≥ 140/90 mmHg had not previously received a medical diagnosis could be the fact that hypertensive disease is silent and, thus, might lead to a lower demand for healthcare services.^7^ The fact that women are more frequently diagnosed and treated has also been pointed out in studies, through the observation that women tend to seek medical services more frequently and therefore have more opportunities for diagnosis, as well as greater adherence to treatment.^3^^,^^7^^,^^8^

A review study^16^ showed that among people with high blood pressure, the proportion of people who were undergoing treatment was low, reaching 29.2% for men and 40.5% for women, while medical follow-up for hypertension among men was 9.8% and among women 16.2%. These findings are in agreement with the present study.

Other PNS analyses have already indicated that only 3% of individuals in the adult population have never had their blood pressure measured and that, therefore, other opportunities for diagnosing high pressure had previously existed.^14^ The PNS has also revealed that in Brazil, in general, 95.4% of the population that sought medical assistance over the preceding 15 days had managed to be seen, and that the National Health System has greatly contributed towards this access to healthcare services. Family healthcare services and health insurance also provide wide coverage for the population. However, the quality of these services needs to be improved, through application of simple measures such as blood pressure measurement at all medical consultations and healthcare services, both public and private.

Other indicators such as definitions for hypertensive patients based on correlating high blood pressure and use of medications have been described in the literature.^10^^,^^11^^,^^19^ In the present study, measured blood pressure ≥ 140/90 mmHg was used because this is the indicator used by WHO to monitor countries, with a view to attainment of the goal of reducing hypertension by 25% between 2015 and 2025.^23^

Among the limitations of the present investigation, this was an epidemiological study using a standardized technique to make three sequential measurements using a digital device. The measurements were made by trained interviewers and not doctors, and the stethoscope method of blood pressure measurement was not used.^24^ Therefore, there may have been differences due to the different methods for casual blood pressure measurement. There have been reports in the literature of peaks in blood pressure at the time of measurement, also known as white coat syndrome, due to anxiety towards blood pressure measurements, thereby possibly resulting in momentary peaks.^25^ Such occurrences were probably minimal, since the measurements were made by researchers and not doctors. Regarding previous diagnoses and use of medication, since this information was self-reported by the interviewees, there may have been differences in their comprehension or memory bias, among other factors.

## CONCLUSION

In 2013, the Global Action Plan for the Prevention and Control of Non-communicable Diseases was approved by the World Health Assembly. The plan included a set of indicators for combating these diseases, and reduction of high blood pressure was among these. The PNS constitutes the baseline for this indicator, which takes into consideration the frequency of the population with blood pressure ≥ 140/90 mmHg.^23^ These results are very important in relation to monitoring overall targets for reducing hypertension. To reach a relative reduction of 25% in the prevalence of high blood pressure, interventions are needed in order to reduce the consumption of salt and saturated fat, as well as to increase the consumption of fruits and vegetables, along with efforts to decrease the incidence of overweight and obesity and to implement monitoring for early detection and treatment for hypertensive individuals.^23^^,^^26^

## References

[1] World Health Organization (2012). Health statistics and information systems. Estimates for 2000-2012. Cause-specific mortality.

[2] World Health Organization (2011). Global status report on noncommunicable diseases 2010.

[3] Passos VMA, Assis TD, Barreto SM (2006). Hipertensão arterial no Brasil estimativa de prevalência a partir de estudos de base populacional [Hypertension in Brazil: estimates from population-based prevalence studies]. Epidemiol Serv Saúde.

[4] Schmidt MI, Duncan BB, Azevedo e Silva G (2011). Chronic non-communicable diseases in Brazil burden and current challenges. Lancet.

[5] Malta DC, Morais OL, Silva JB (2011). Apresentação do plano de ações estratégicas para o enfrentamento das doenças crônicas não transmissíveis no Brasil, 2011 a 2022 [Presentation of the strategic action plan for coping with chronic diseases in Brazil from 2011 to 2022]. Epidemiol Serv Saúde.

[6] Centers for Disease Control and Prevention Behavioral Risk Factor Surveillance System. National Center for Chronic Disease Prevention and Health Promotion.

[7] Lima-Costa MF, Peixoto SV, Firmo JOA (2004). Validade da hipertensão arterial auto-referida e seus determinantes (projeto Bambuí) [Validity of self-reported hypertension and its determinants (the Bambuí study)]. Rev Saúde Pública.

[8] Andrade SSCA, Malta DC, Iser BM, Sampaio PC, Moura L (2014). Prevalência da hipertensão arterial autorreferida nas capitais brasileiras em 2011 e análise de sua tendência no período de 2006 a 2011 [Prevalence of self-reported arterial hypertension in Brazilian capitals in 2011 and analysis of its trends in the period between 2006 and 2011]. Rev Bras Epidemiol.

[9] Brandão A (2010). Hipertensão conceituação, epidemiologia e prevenção primária. Rev Bras Hipertens.

[10] Chor D, Pinho Ribeiro AL, Sá Carvalho M (2015). Prevalence, Awareness, Treatment and Influence of Socioeconomic Variables on Control of High Blood Pressure Results of the ELSA-Brasil Study. PLoS One.

[11] Lotufo PA (2015). Melhorando o controle da hipertensão arterial Dados iniciais do Estudo Longitudinal de Saúde do Adulto (ELSA-Brasil). Diagn Tratamento.

[12] Brasil. Ministério da Saúde.Instituto Brasileiro de Geografia e Estatística. Ministério do Planejamento, Orçamento e Gestão (2014). Pesquisa Nacional de Saúde: 2013. Percepção do estado de saúde, estilos de vida e doenças crônicas. Brasil, grandes regiões e unidades da federação.

[13] Souza-Júnior PRB, Freitas MPS, Antonaci GA, Szwarcwald CL (2015). Desenho da amostra da Pesquisa Nacional de Saúde 2013 [Sampling Design for the National Health Survey, 2013]. Epidemiol Serv Saúde.

[14] Brasil. Ministério da Saúde. Instituto Brasileiro de Geografia e Estatística (2015). Pesquisa Nacional de Saúde: 2013. Ciclos de Vida. Brasil e grandes regiões.

[15] Cooper R, Puras A, Tracy J (1997). Evaluation of an electronic blood pressure device for epidemiological studies. Blood Press Monit.

[16] Pereira M, Lunet N, Azevedo A, Barros H (2009). Differences in prevalence, awareness, treatment and control of hypertension between developing and developed countries. J Hypertens.

[17] Lessa I, Magalhães L, Araújo MJ (2006). Hipertensão arterial na população adulta de Salvador (BA) - Brasil [Arterial hypertension in the adult population of Salvador (BA) - Brazil]. Arq Bras Cardiol.

[18] Firmo JOA, Uchôa E, Lima-Costa MF (2004). Projeto Bambuí fatores associados ao conhecimento da condição de hipertenso entre idosos [The Bambuí Health and Aging Study (BHAS): factors associated with awareness of hypertension among older adults].. Cad Saúde Pública.

[19] Barreto SM, Passos VMA, Firmo JOA (2001). Hypertension and clustering of cardiovascular risk factors in a community in Southeast Brazil -- The Bambuí Health and Ageing Study. Arq Bras Cardiol.

[20] Paulucci TD, Velasquez-Mendelez G, Bernal RIT, Lana FF, Malta DC (2014). Análise do cuidado dispensado a portadores de hipertensão arterial em Belo Horizonte, segundo inquérito telefônico [Analysis of care given to patients with hypertension in Belo Horizonte, according to telephone survey]. Rev Bras Epidemiol.

[21] Brasil. Instituto Brasileiro de Geografia e Estatística (2010). Censo demográfico 2010.

[22] Nogueira D, Faerstein E, Coeli CM (2010). Reconhecimento, tratamento e controle da hipertensão arterial estudo Pró-Saúde, Brasil [Awareness, treatment, and control of arterial hypertension: Pró-Saúde study, Brazil]. Rev Panam Salud Pública.

[23] World Health Organization (2013). Noncommunicable diseases and mental health. Global action plan for the prevention and control of NCDs 2013-2020.

[24] Mancia G, Parati G (2004). Office compared with ambulatory blood pressure in assessing response to antihypertensive treatment a meta-analysis. J Hypertens.

[25] Nascimento LR, Molina MC, Faria CP, Cunha RS, Mill JG (2013). Reprodutibilidade da pressão arterial medida no ELSA-Brasil com a monitorização pressórica de 24h [Reproducibility of arterial pressure measured in the ELSA-Brasil with 24-hour pressure monitoring]. Rev Saúde Pública.

[26] Malta DC, Silva JB (2013). O plano de ações estratégicas para o enfrentamento das doenças crônicas não transmissíveis no Brasil e a definição das metas globais para o enfrentamento dessas doenças até 2025: uma revisão [Brazilian strategic action plan to combat chronic non-communicable diseases and the global targets set to confront these diseases by 2025: a review]. Epidemiol Serv Saúde.

